# Detectability of Head and Neck Cancer via New Computed Tomography Reconstruction Tools including Iterative Reconstruction and Metal Artifact Reduction

**DOI:** 10.3390/diagnostics11112154

**Published:** 2021-11-21

**Authors:** Daniel Troeltzsch, Seyd Shnayien, Max Heiland, Kilian Kreutzer, Jan-Dirk Raguse, Bernd Hamm, Stefan M. Niehues

**Affiliations:** 1 Department of Oral and Maxillofacial Surgery, Charité-Universitätsmedizin Berlin, Charitéplatz 1, 10117 Berlin, Germany; max.heiland@charite.de (M.H.); kilian.kreutzer@charite.de (K.K.); 2 Department of Radiology, Charité-Universitätsmedizin Berlin, Charitéplatz 1, 10117 Berlin, Germany; seyd.shnayien@charite.de (S.S.); bernd.hamm@charite.de (B.H.); stefan.niehues@charite.de (S.M.N.); 3 Department of Oral and Maxillofacial Surgery, Fachklinik Hornheide, 48157 Münster, Germany; Jan-Dirk.Raguse@fachklinik-hornheide.de

**Keywords:** cancer diagnostics, head and neck cancer, OSCC, SCC, iterative reconstruction, computed tomography, metal artifact reduction

## Abstract

State-of-the-art technology in Computed Tomography (CT) includes iterative reconstruction algorithms (IR) and metal artefact reduction (MAR) techniques. The objective of the study is to show the benefits of this technology for the detection of primary and recurrent head and neck cancer. A total of 131 patients underwent contrast-enhanced CT for diagnosis of primary and recurrent Head and Neck cancer; 110 patients were included. All scans were reconstructed using iterative reconstruction, and metal artifact reduction was applied when indicated. Tumor detectability was evaluated dichotomously. Histopathological findings were used as a standard of reference. Data were analyzed retrospectively, statistics was performed through diagnostic test characteristics. State-of-the-art Head and Neck CT showed a sensitivity of 0.83 (95% CI; 0.61–0.95) with 0.93 specificity (95% CI; 0.84–0.98) for primary tumor detection. Recurrent tumors were identified with a 0.94 sensitivity (95% CI; 0.71–0.99) and 0.93 specificity (95% CI; 0.84–0.98) in this study. Conclusion: State-of-the-art reconstruction tools improve the diagnostic quality of Head and Neck CT, especially for recurrent tumor detection, compared with data published for standard CT. IR and MAR are easily implemented in routine clinical settings and improve image evaluation by reducing artifacts and image noise while lowering radiation exposure.

## 1. Introduction

According to the WHO World Cancer Report, cancer of the head and neck (H&N) is the seventh most frequently occurring cancer worldwide [1]. The GLOBOCAN database indicates that over 700,000 new cases of lip, oral, oropharyngeal, hypopharyngeal, and nasopharyngeal cancer, as well as cancer of the salivary glands combined are reported annually across the globe [2]. Oral squamous cell carcinoma is the most common oral malignancy, accounting for 80–90% of cases [3].

Patient outcomes can be improved by early detection and effective treatment before cancer spreads to lymph nodes or distant organs. Mortality depends on the tumor stage at diagnosis. With approximately 145 deaths per 100,000 inhabitants worldwide, mortality rates of HNSCC are high [4].

Along with clinical and histopathological assessment, pretreatment imaging plays a major role in the management of patients with H&N cancer [5]. Contrast-enhanced computed tomography (CT) is broadly available and considered the main imaging technique for the initial diagnosis and evaluation of tumor extent [6]. The limitations of CT are the frequent occurrence of image artifacts and the substantial exposure of patients to ionizing radiation. Image artifacts originate from metallic dental restorations (metal artifacts). Metal artifacts majorly influence tumor detection in the head and neck region [7]. As patients become older and the prevalence of metal restorations increases, in the majority of cases, cancer detection is challenged. Misdiagnosis or delayed diagnosis may occur due to the close relationship between restorations and affected anatomical structures in the oral cavity, particularly near the root of the tongue and the palate.

Radiation exposure is regarded in the context of the cumulative dose of radiation from medical imaging procedures increased over a lifespan [8]. CT scans and nuclear imaging cause about three-fourths of the cumulative effective dose [8]. The diagnostic reference level for H&N CT scans in Germany, given as a dose-length product (DLP), is as high as 330 mGy*cm [9]. The implications of radiation exposure are even more relevant in an ageing society.

State-of-the-art CT technology includes iterative reconstruction (IR) for lower radiation exposure and metal artifact reduction (MAR) techniques. Therefore, major limitations of CT for head and neck cancer are addressed. In the past few years, contrast-enhanced CT has been improved by the advent of new reconstruction tools, such as iterative reconstruction and techniques of artifact reduction, like Single-Energy Metal Artifact Reduction (SEMAR) or dual-energy imaging [10]. These methods allow CT images with improved quality and resolution and less noise, while at the same time significantly lowering radiation exposure [11]. The technical knowledge behind iterative reconstruction (IR) has already been developed in the 1970s [12], but the computational demand compared to other analytical methods was too high. It was not until recent years that the advances in computer technology made IR broadly available. The standard reconstruction algorithm, Filtered Back Projection (FBP), is the still the most common reconstruction tool in CT [13]. However, it causes a decrease in image quality as the tube current is reduced for less radiation. With iterative reconstruction, image quality improves with lower radiation exposure [11]. Data are processed in an ongoing loop and compared to “ideal” models to improve the CT image step-by-step [14].

To reduce metal artifacts due to dental hardware, new techniques of metal artifact reduction, including data segmentation, forward projection, and interpolation have been implemented. This significantly improved image quality and helped overcome the limitations of CT in this region [15].

There is no recent data on the performance of Head and Neck Squamous Cell Carcinoma (HNSCC) detection using state-of-the-art CT technology in combination with new imaging tools. We conducted this study to investigate the actual diagnostic value of current CT techniques in a real clinical setting. Is there a similar or even higher diagnostic value of recent CT techniques in comparison to CT without IR and MAR? All findings are correlated with clinical and histological data.

## 2. Materials and Methods

This clinical study is in compliance with the Helsinki Declaration and was approved by our local ethics committee (Approval number EA4/066/14). Informed consent forms were signed.

### 2.1. Study Population

A total of 131 consecutive patients examined by H&N CT between 24 January 2014 and 29 January 2018 were considered for study inclusion. A total of 110 patients who underwent CT for either diagnosis of clinically suspected HNSCC (*n* = 40) or routine HNSCC follow-up (*n* = 70) met the criteria for study inclusion. CT datasets were evaluated for tumor detectability. Inclusion criteria for patients with malignancies were: entity of HNSCC and histopathological confirmation of HNSCC. For tumor-free patients, a follow-up period of at least 10 months had to be respected if no biopsy was obtained.

**Figure 1:**
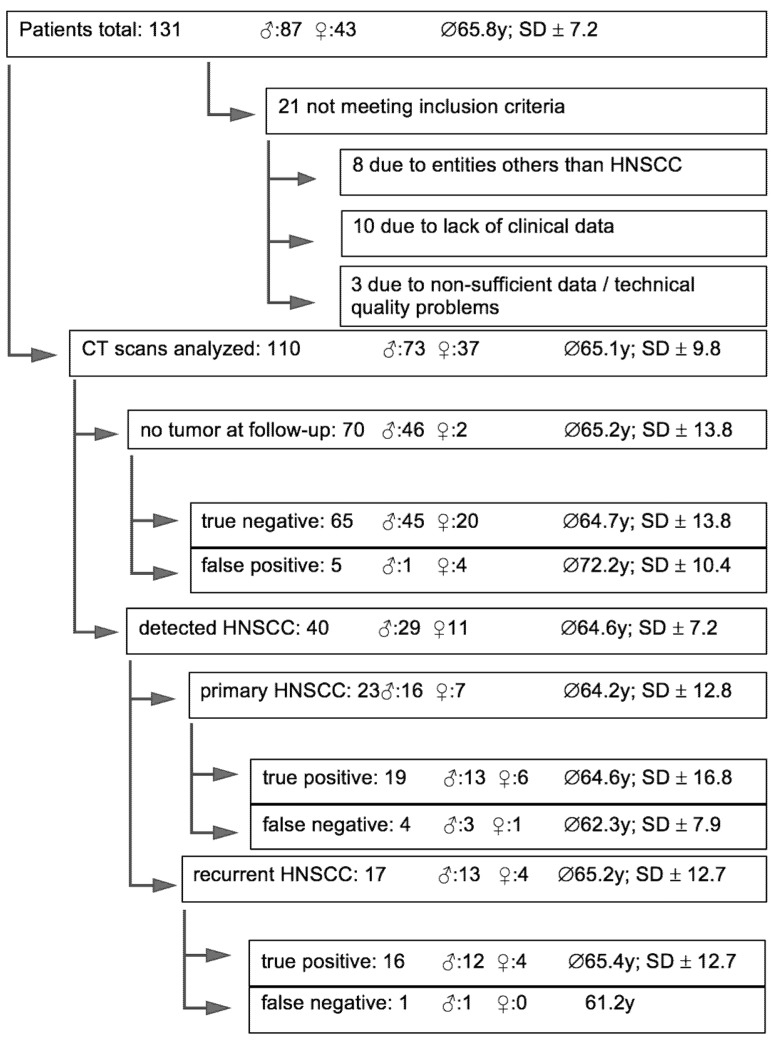
Patient inclusion and exclusion; demographic data; and distribution of Head and Neck Squamous Cell Carcinoma (HNSCC); CT findings.

Study patients were divided into three groups: initial diagnosis of HNSCC (I), recurrent HNSCC (II), and no HNSCC at the time of investigation (III). For all patients, full clinical, histological, and radiological data had to be available. Additional exclusion criteria were a pre-known residual tumor and a second imaging technique available for the same data set. A total of 110 of 131 patients met the inclusion criteria; 21 patients were excluded. In 10 cases, full clinical data were not available, 8 patients presented entities others than HNSCC; in 2 patients, CT scans could not be analyzed independently due to a pre-known residual tumor; in one case, an MRI scan was simultaneously examined (Figure 1).

### 2.2. CT Protocol

All CT examinations were performed on an 80-slice CT scanner (Aquilion PRIME, Canon Medical Systems, Otawara, Japan). A biphasic contrast administration protocol was used. The first bolus of a 50 mL contrast agent (Imeron 400 MCT, Bracco) was followed by a 50 mL flush of saline. After 55 sec, the second bolus of 30 mL contrast agent was followed by a 40 mL saline flush to differentiate arterial vessels in the H&N area. After 18 s, a helical scan covering the entire neck from the frontal sinus to the aortic arch was acquired. Routinely, iterative reconstruction (AIDR3D, standard level) was applied. In those cases, where metal dental restorations were detected on the scout view, an additional dataset using Single-Energy Metal Artifact Reduction (SEMAR) was reconstructed. Scan parameters were: Auto-kV and Auto-mA with an SD of 7.5, 0.75 sec rotation time, 80 × 0.5 mm collimation, and pitch factor of 0.813.

### 2.3. Data Assessment

All CT datasets were analyzed by two radiologists of a maximum care hospital in a consensus reading session. Tumor detectability was further evaluated dichotomously. Histological examinations were carried out by two independent pathologists of the hospital’s pathology department if a tumor was clinically and/or radiologically suspected.

The primary item to be evaluated was the existence of a tumor.

### 2.4. Statistical Analysis

CT findings were correlated with histopathological results and/or clinical findings after a minimum follow-up of 10 months if no HNSCC was detected. Statistics were retrospectively calculated based on whether radiological findings were accurate.

Sensitivity, specificity, positive predictive value (PPV), negative predictive value (NPV), and accuracy of CT scans with techniques of dose reduction and SEMAR were assessed through diagnostic test characteristics.

Ninety-five percent confidence intervals (95% CIs) were derived for all diagnostic accuracy parameters. Statistical analysis was performed using IBM SPSS Statistics 24.

## 3. Results

### 3.1. Patients

Of the 110 patients included in our study, 73 were men (66%) and 37 women (34%). They had a mean age of 65.2 years (± 13.8 SD) with a range of 46 to 95 years.

The distribution of tumor localizations at the initial diagnosis, as well as the frequency of each location in primary (I), recurrent (II), and no tumor patients (III) is given in Table 1. The results of T-tumor staging (eighth edition of UICC TNM Classification) at initial diagnosis are summarized in Table 2.

### 3.2. CT Findings

Malignancies were radiologically identified in 40 study patients, among them 23 cases of primarily diagnosed HNSCC and 17 patients with a recurrent tumor. A total of five HNSCC were misdiagnosed as benign tissue. Three of the four false-negative patients with primary HNSCC (75%) had tumor stage T1 and one stage T2. Seventy patients had no HNSCC at the time of CT examination. Five of the patients without HNSCC based on clinical and histological findings were false-positive. Detailed data are presented in Table 3.

Group III (no HNSCC) was used on top of both groups I (primary HNSCC) and II (recurrent HNSCC).

### 3.3. Technical Results

**Figure 2:**
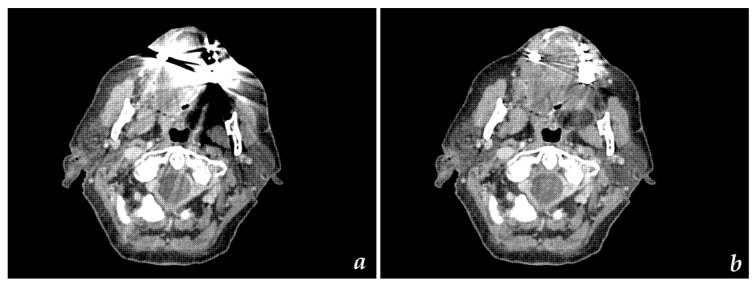
Axial CT images with (**a**) AIDR reconstruction and (**b**) SEMAR reconstruction illustrating greater homogeneity of the SEMAR dataset and significant reduction of metal artifacts.

**Figure 3:**
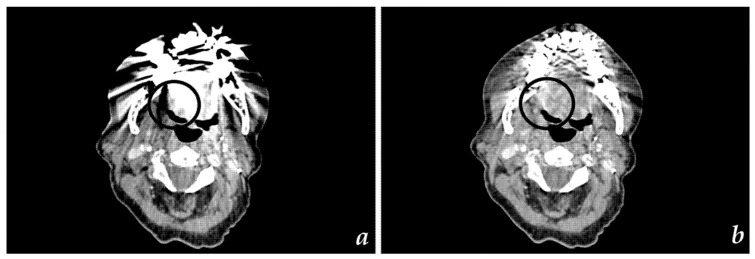
Axial CT images of (**a**) recurrent HNSCC of tongue edge (highlighted) without SEMAR reconstruction and (**b**) recurrent HNSCC of tongue edge (highlighted) with SEMAR reconstruction.

All diagnostic CT scans were uneventful in all patients. Images were generated using iterative reconstruction (Figure 2a,b). The total mean dose-length product (DLP) of H&N CT scans with iterative reconstruction was 219 mGy*cm in this study. If metal dental hardware was scouted, SEMAR was successfully applied to all relevant images, significantly reducing metal artifacts (Figure 2b and Figure 3b). The median additional reconstruction time required for a SEMAR dataset was 224 sec.

### 3.4. Detectability of Primary (I) and Recurrent HNSCC (II) via CT

Tumor detectability via CT, reconstructed with AIDR 3D and SEMAR, for patients with primary and recurrent HNSCC is given in Table 4.

## 4. Discussion

Our study provides important information regarding the performance of CT in the head and neck area. CT scans with state-of-the-art reconstruction tools, such as SEMAR and iterative reconstruction, show superior performance compared to standard CT diagnostics in regard to primary and recurrent tumor detection in the head and neck area.

Effective tumor treatment depends on early tumor detection with a high performance of clinical and radiological examinations. The extent of primary and recurrent tumor lesions and the potential infiltration of adjacent anatomical structures were assessed for treatment planning and prognosis of the disease. Radiologic examinations were restricted by the ALADA (as low as diagnostically applicable) principle. It allowed for the identification of patients for whom surgery may not be the first option, for example, if the tumor was already invading the internal carotid artery.

Especially in the elderly population, to which most of our patients diagnosed with head and neck cancer belonged (average age of about 64 years in this study), dental restorations are an increasing issue, causing metal artifacts in CT scans.

To the best of our knowledge, no study published on the use of CT scans in the detection of head and neck cancer included iterative reconstruction as a state-of-the-art technique or a combination of both metal artifact reduction and iterative reconstruction in a real clinical setting. Little scientific data are available, while these techniques are increasingly being used in clinical routines worldwide.

CT scans are the most commonly used modality for tumor detection. CT scans are relatively cheap, fast, and readily available. Therefore, it is important to understand the evolution of this technique with its benefits und limitations.

A review of the literature including more than 50 scientific studies on the role of imaging in H&N cancer was published by Blatt et al. in 2016 [6]. Most of the studies discussed in this review investigated functional techniques, such as FDG-PET/CT, FDG-PET/MRI, and FAMT-PET/CT; and no study evaluated what was used in the daily clinical routine: contrast-enhanced CT or advances in H&N CT resulting from the advent of new reconstruction and post-processing tools. For almost two decades, CT and Magnetic Resonance Imaging (MRI) have been considered the gold standard for primary diagnosis and evaluation of local tumor spread [16]. Recent tools that have contributed to the improvement of CT image quality include noise reduction and metal artifact reduction. At the same time, there are new options to lower radiation doses. Our study shows that these techniques are highly sensitive and specific in the head and neck area, especially in terms of detecting recurrent HNSCC. One possible explanation for the better performance of modern CT techniques in recurrent tumors is that there might be less interference within recurrence imaging as there is simply less foreign material left. Often in patients with local recurrences, teeth, implants, and dental bridges that are not worth preserving have already been removed in the course of primary resection of the jaw or will be removed to avoid complications such as osteoradionecrosis if adjuvant radiotherapy is likely. According to our experience, SEMAR reduces metal artifacts very well, especially if dental hardware keeps a little distance from the area to be examined. The area predestined for local recurrences is now much more evaluable. This could explain why the new CT technology performs better in recurrence tumor detection compared to primary tumor detection. Most published data on HNSCC detection refer to H&N CT without iterative reconstruction (IR), metal artifact reduction (MAR), or other new processing tools. IR, MAR, and various other dose reduction techniques have started to enter clinical routines in recent years, and their use is increasing worldwide.

Studies investigating the detectability of primary H&N cancer with standard CT report sensitivities of 0.52 [16], 0.55 [17], and 0.68 with a specificity of 0.69 [18].

In a review of the literature by Palasz and colleagues, which also included more recent studies, sensitivity of H&N CT was found to range from 0.42 to 0.82 with 0.82–1 specificity for primary tumor detection [19].

Our study outperformed the average results published on former studies. It showed a higher sensitivity of 0.83 with 0.93 specificity for the primary detection of HNSCC.

For the detection of recurrent HNSCC, sensitivity was even higher with 0.94.

The achieved sensitivities and specificities for H&N CT with IR and MAR are significantly better than that reported for CT without IR and MAR.

For the detection of recurrent oropharyngeal tumors by CT, studies report sensitivities ranging from 0.50 [19], to 0.68 [18] up to 1 [16]. Note, though that a sensitivity level of 1 was achieved in an analysis of only nine patients.

MRI is an important alternative imaging modality for the diagnostic evaluation of patients with H&N tumors, including determination of local tumor extent and detection of bone invasion. A general recommendation in favor of either MRI or CT has not been given so far [20,21,22]. An important advantage of MRI is that it does not use ionizing radiation. Important disadvantages of MRI include long examination times, during which patients must not move and image deterioration caused by metallic restorations. Furthermore, gadolinium-based contrast agents have recently been found to accumulate in the brain after repeat administration [23]. Therefore, special recommendations have been issued for the use of gadolinium-based contrast agents for MRI [24].

Our data also show that CT is superior to PET/CT in detecting recurrent HNSCC. In a systematic review and meta-analysis of Sheikhbahaei et al., follow-up FDG PET or PET/CT in patients with head and neck cancer had 0.92 sensitivity (95% CI, 0.90–0.94) and 0.87 specificity (95% CI, 0.82–0.91) compared with 0.94 and 0.93 for CT in our study [25]. Generally, PET/CT is more expensive and not as widely available as regular CT scanning. For special indications, such as cervical lymph node staging, 18F-FDG PET/CT is more accurate than regular H&N CT [26]. PET/CT is also recommended for the detection of bone marrow invasion.

State-of-the-art processing tools for H&N CT reduce image noise, resulting in consistent or better image quality while at the same time allowing scanning with less radiation exposure. Images in our study were of high quality, allowing initial diagnostic evaluation and detection of recurrent HNSCC.

Low radiation exposure is important, as patients have to undergo CT scans on a regular basis in their tumor follow-ups. To assess the exposition to radiation, the dose-length product (DLP) of CT scans were measured.

The DLP of CT scans in this study was 219 mGy*cm versus 330 mGy*cm for comparable CT scans acquired without iterative reconstruction [9]. This corresponds to 34% dose reduction while image quality was constant or even improved.

A similar observation was made for chest CT, for which a 50% reduction of radiation exposure was achieved with iterative reconstruction, along with an improvement in image quality [27].

This study has some limitations. Metal artifact reduction (MAR) and iterative construction tools for CT are not standardized and may differ from one manufacturer to the next. Therefore, the results achieved here in terms of tumor detection and radiation exposure may not be directly applicable to other settings. The AIDR3D iterative reconstruction package from Canon Medical was used, which may differ from tools such as ADMIRE (Siemens) or ASIR (GE). Another limitation is that data were analyzed retrospectively.

Meaning of this study:

With this study, we want to update colleagues on the benefits and limitations of recently evolved and broadly available CT techniques. We also hope to encourage others to take advantage of them. Compared to published data, state-of-the-art reconstruction tools and metal artifact reduction improve the performance of contrast-enhanced CT scans for primary and recurrent HNSCC detection. These techniques are easily implemented in daily clinical routines where an increasing number of head and neck cancer patients present with dental metal hardware. At the same time, IR and MAR reduce radiation exposure by one-third, strengthening the role of CT compared with MRI in these patients.

Further research should be carried out in a multi-centric manner with a greater number of patients in each cohort. It would also be interesting to evaluate accuracy parameters for different intraoral locations by itself and with the usage of lip holders and cheek pieces to separate anatomical structures and dental hardware.

## Figures and Tables

**Table 1:** Distribution of tumor localizations at initial diagnosis.

Tumor Localization at Initial Diagnosis	Frequency in Total (*n*)	Percentage	Frequency (*n*) and Percentage of Locations in Primary (I), Recurrent (II) and no Tumor (III)
Mouth floor	35	31.8%	I: 9 (25.7%) II: 5 (14.3%) III: 21 (60.0%)
Oropharynx	22	20.0%	I: 4 (18.2%) II: 3 (13.6%) III: 15 (68.2%)
(Alveolar ridge of upper/lower jaw/buccal mucosa	23	20.9%	I: 6 (26.1%) II: 2 (8.7%) III: 15 (65.2%)
Edge of tongue	20	18.2%	I: 3 (15.0%) II: 3 (15.0%) III: 14 (70.0%)
Laryngopharynx	5	4.6%	I: 0 (0.0%) II: 3 (60.0%) III: 2 (40.0%)
Nasopharynx	2	1.8%	I: 1 (50.0%) II: 0 (0.0%) III: 1 (50.0%)
Others	3	2.7%	I: 0 (0.0%) II: 1 (33.3%) III: 2 (66.7%)

**Table 2:** Distribution of T- stages at initial diagnosis (based on eighth UICC TNM Classification).

T-Stage	Number *n*	Percentage %
T1	36	32.7
T2	33	30.0
T3	11	10.0
T4	24	21.8
Tx	6	5.5

**Table 3:** Distribution of diagnostic findings (as compared to pathology) by group.

Patient Group	Number of Patients	True Positive	False Negative	True Negative	False Positive
Primary HNSCC (I)	23	19	4	0	0
Recurrent HNSCC (II)	17	16	1	0	0
No HNSCC (III)	70	0	0	65	5

**Table 4:** Detectability of primary and recurrent Head and Neck Squamous Cell Carcinoma (HNSCC) with state-of-the-art CT technology in comparison to ranges of standard CT values from literature.

Statistical Results	Primary Data Primary HNSCC Recurrent HNSCC		Comparative Range from Literature Primary HNSCC Recurrent HNSCC	
Sensitivity	0.83 (95% CI; 0.61–0.95)	0.94 (95% CI; 0.71–0.99)	0.42–0.82 [16,17,18]	0.50 [19]–0.68 [16,17,18]
Specificity	0.93 (95% CI; 0.84–0.98)	0.93 (95% CI; 0.84–0.98)	0.69–1 [18,19]	0.88 [19]
PPV	0.79 (95% CI; 0.62–0.90)	0.76 (95% CI; 0.58–0.88)		
NPV	0.94 (95% CI; 0.87–0.98)	0.98 (95% CI; 0.91–0.99)		
Accuracy	0.90 (95% CI; 0.82–0.95)	0.93 (95% CI; 0.86–0.97)		

## Data Availability

The data presented in this study are available in the article itself (i.e., Table 3).
